# Re-Vaccinations in the Prussian Army in 1847

**Published:** 1849-01

**Authors:** 


*Re-Vaccinations in the Prussian Army in* 1847.—The total number of men vaccinated was 43,264; the number of those who bore marks of a former vaccination in a decided manner, 34,264; ditto with the marks not very distinct, 6,405; ditto with marks not visible at all, 2,927. The vaccine virus developed itself satisfactorily in 25,544; very irregulary in 7,425; and not at all in 10,527. The vaccinations which had yielded no results were repeated; they acted in 2,718; and failed entirely in 8,952 cases. In consequence of the present vaccination, there were developed from one to five vaccine pustules upon 13,295; from six to ten upon 8,164; from eleven to twenty upon 5,767; from twenty-one to thirty upon 1,036 of the men. Amongst those who were vaccinated in the year 1847, there was within the same year, no case of varicella, none of actual small-pox, and only one of chicken-pox. The lymph was obtained from vaccinated children or grown-up persons. It is remarkable that amongst those who were subject to re-vaccination there were several who had had the small-pox before, yet upon whom the vaccine matter produced the usual pustules.—*Lancet.*

